# How to face the aging world – lessons from dementia research

**DOI:** 10.3325/cmj.2020.61.139

**Published:** 2020-04

**Authors:** Dinko Mitrečić, Dražen Juraj Petrović, Paula Stančin, Jasmina Isaković, Barbara Zavan, Gerardo Tricarico, Mirjana Kujundžić Tiljak, Monica Di Luca

**Affiliations:** 1 Laboratory for Stem Cells, Department of Histology and Embryology, Croatian Institute for Brain Research, University of Zagreb School of Medicine, Zagreb, Croatia; 2 Genos d.o.o., Zagreb, Croatia; 3 Omnion Research International d.o.o., Zagreb, Croatia; 4 Department of Medical Sciences, University of Ferrara, Ferrara, Italy; 5 Department of Health Sciences, University of Eastern Piedmont Amedeo Avogadro, Novara, Italy; 6 Andrija Štampar School of Public Health University of Zagreb, School of Medicine, Zagreb, Croatia; 7 European Brain Council, Brussels, Belgium

## Abstract

A continuous rise in life expectancy has led to an increase in the number of senior citizens, now amounting to a fifth of the global population, and to a dramatic increase in the prevalence of diseases of the elderly. This review discusses the threat of dementia, a disease that imposes enormous financial burden on health systems and warrants efficient therapeutic solutions. What we learned from numerous failed clinical trials is that we have to immediately take into account two major elements: early detection of dementia, much before the onset of symptoms, and personalized (precision) medicine treatment approach. We also discuss some of the most promising therapeutic directions, including stem cells, exosomes, electromagnetic fields, and ozone.

World Health Organization (WHO) proclaimed 2020-2030 “the decade of healthy aging” (1). By dedicating a whole decade to aging, the WHO wants to draw attention to the steep increase in human life expectancy in the last 100 years and a huge challenge that this phenomenon presents for humanity. On average, every 10 years, life expectancy increases for 2-4 years, which means that in 1950s the average life expectancy was 45 years, in 1980s 60 years, and now it is 75 years (2). The decade of healthy aging is our attempt to tackle the approaching problems: our planet is changing dramatically, and the current proportion of the population above 60 years (17%) will double in only thirty to forty years. The fact that in the next 30 years every third person in the world will fall in the category of senior citizen urges the adoption of completely new strategies in medical, social, and technological fields (3).

Besides vascular diseases and two major causes of death – heart and brain ischemia – neurodegeneration with consequent dementia presents a health condition with the most significant health burden. Alzheimer’s disease (AD) is a growing public health concern worldwide. In Europe, an estimated 10.5 million people live with dementia, and this number is expected to increase by 2050 to 18.7 million. Since only coordinated action can stop such diseases, European countries united under the umbrella of the European Brain Council (EBC). The EBC gathers scientific societies, patient organizations, professional societies, and industry partners. Its main mission is to promote brain research and all the activities that can bring benefits in this field. The major goal is to improve the lives of the estimated 179 million Europeans living with brain diseases. EBC offers its expertise and networks to foster cooperation between its member organizations, promoting and improving cooperation among scientists and various stakeholders in the field, including industry and general society. Notably, EBC emphasizes the importance of raising awareness and encouraging education on the brain and the consequences of brain diseases.

## Dementia is still not a sufficiently recognized threat

While other chronic diseases of the elderly mostly cause recognizable symptoms, pain, impairment, and discomfort, Alzheimer`s and other types of dementia are rarely correctly recognized by the general public owing to their specific symptoms. The Croatian Brain Council developed a questionnaire with only one question: “When entering mature years of your life, it is expected that you will face some health problems. Which of them are you scared the most?” On the top of the participants’ list there were various cancers (breast cancer for 66 participants, gastrointestinal cancers for 63, and lung cancer for 40) even though the majority of neoplastic diseases are nowadays successfully cured. Dementia was mentioned by only 11 out of 200 participants despite the fact that it can have the most humiliating consequences for a human being and despite the lack of efficient drugs to treat its fully developed form. It was recognized as slightly less important than “joint problems” (16 participants) and slightly more important than “sexual impotence” (8 participants). This finding is in strong disagreement with the fact that, apart from dramatic gradual disintegration of the person, the financial burden for the family is much higher than for any other human disease. Annual costs vary for different levels of care and countries, but are still enormous: from the minimum of $ 5000 in Croatia to more than $ 120 000 in the USA (4). The EBC demonstrated that in Europe the total cost of brain diseases on a yearly basis amounted to € 900 billion; for dementia only, the cost was € 22 000 per patient per year. When we know that the number of seniors will double in the next decades, when we know the number of caregivers dedicated to every case, and when we know that recent studies failed to offer new therapeutic options, it is obvious that the urgent action is needed.

## Early diagnostics and personalized approach are the key points in the treatment of dementia

Thus far, the majority of clinical trials for new dementia drugs have failed. This can be attributed to the brain's innate complexity and a high level of individuality. Brain diseases are multifactorial, with a very complex background and variable onset and progression. Moreover, the obvious symptoms usually appear only when the disease enters the last stage of progression. Therefore, we recognize two major strategic directions for improving dementia treatment: 1) detection of the pathological process before the onset of symptoms and 2) personalized treatment approach (5). Personalized approach can be especially effective when it comes to modifying the following parameters: insulin resistance (6), increased homocysteine levels (7), lack of exercise (8), lack of sleep (9), and various toxic factors (10) (Table 1). These factors impair the function of a critical number of neurons, but in a very individualized way. A cheap but very effective way to treat neurodegenerative diseases may be intermittent fasting, ie, fasting regimens with recurring fasting periods lasting 24-48 hours, and time-restricted feeding, which consists of daily fasting periods lasting 12-20 hours (11). Thus, the adjustment of these parameters can successfully reverse cognitive decline (12-15). Another strategy relies on repurposing the existing drugs for the therapy of neurodegenerative diseases with a personalized twist. Trazodone hydrochloride, a licensed anti-depressant, and dibenzoylmethane, a substance found in licorice, restored protein synthesis rates in prion-diseased and tauopathy frontotemporal dementia mice with established disease (16). De Coco et al (17) have shown that aged mice treated with nucleoside reverse transcriptase inhibitor lamivudine had down-regulated interferon 1 activation and age-associated inflammation in several tissues. Zhang et al (18) have shown that amyloid beta oligomer can hijack norepinephrine-elicited signaling through alpha adrenergic receptor 2A (α_2A_AR) to activate pathogenic glycogen synthase kinase 3 beta/tau cascade. α_2A_AR blockers, such as idazoxan, are already used for the treatment of other disorders, and repurposing these drugs could be a potentially effective, readily available strategy for AD treatment (18). The mutations of glucocerebrosidase gene, *GBA1*, are the most important risk factor for Parkinson disease (PD) (19). *In vitro* and *in vivo* studies have reported that β-glucocerebrosidase enzyme activity can be increased, and α-synuclein levels reduced, by ambroxol (19). A recent non-randomized, non-controlled study has suggested that ambroxol therapy was safe and well tolerated and that ambroxol penetrated the blood brain barrier (BBB) well (19). Ambroxol is a promising treatment modality, but a placebo-controlled clinical trial is necessary to examine if it has any effect on the natural progression of PD. All these drugs show huge individual differences in every tested patient. Understanding the basis of this variability will help to improve the general efficacy of future therapeutic approaches.

**Table 1:** Major promising strategies for the treatment of neurodegenerative disorders

Repurposed drugs	Reference
Trazodone hydrochloride and dibenzoylmethane	(16)
Lamivudine	(17)
Idazoxan	(18)
Ambroxol	(19)
**Physiological parameters suitable for personalization**	
Insulin resistance	(6)
Vitamin B12 and homocysteine	(7)
Physical activity	(8)
Sleep	(9)
Intermittent fasting and time restricted feeding	(11)
Individualized multifactorial protocols	(12-15)
**Exosomes**	(37,38)
**Electromagnetic fields**	(39-51)
**Ozone**	(57)

Apart from personalized approach, here we stress the need for early diagnostics. Some molecules have already revealed a clear benefit in the preclinical set up, while some are still entering clinical applications. Cerebrospinal fluid (CSF) biomarkers include amyloid β (Aβ42, Aβ42/40), total tau (T-tau), and phosphorylated tau (P-tau 181). These biomarkers have a high diagnostic accuracy in earlier disease stages. Many studies have shown that patients with Aβ have elevated levels of T-tau and P-tau protein and decreased levels of β-amyloid (20). Other most promising biomarkers in the CSF include TREM2, a transmembrane receptor that is expressed by microglia (21) and visine-like protein 1, a neuronal calcium-sensing protein that participates in neuroprotection and neurotoxic processes in the brain. It is a marker of neural damage and it correlates well with T-tau and P-tau 181 (22). Neurogranin (NGRN) is a postsynaptic protein that is expressed mainly in the cortical regions and is associated with cognition. Reduced NGRN levels are found in the cortex and hippocampal region of the brain, which are most affected by AD. NGRN can be used to predict the progression of cognitive deficit and can indicate the loss of synapses (23). The β-site of APP cleaving enzyme 1 (BACE-1) is a β-secretase that is important for the cleavage of APP protein, while the resulting peptides aggregate and form extracellular plaques. The levels of this enzyme are elevated in people with AD compared with healthy controls and patients with other forms of dementia (24). Kallikrein-8 (KLK8) is a protease (neuropsin) up-regulated in the hippocampus of patients with early dementia. Short-term KLK8 inhibition in moderate stage of the disease mitigated the features of Alzheimer (25).

The major obstacle in recognizing these biomarkers is their poor or not existing availability in blood. To overcome the need for CSF collection or other invasive and complex methods, several new concepts have been developed. Some of them are based on stem cell technology, which continues to offer new treatment options in biomedicine. Apart from being used in research (26) and in therapeutic applications (27,28), stem cells also offer new possibilities in early diagnostics. One of the most innovative approaches using stem cells is the “Check My Brain” platform, owned by Omnion Research International (29). It offers a simple procedure of transformation of hairs into neurons, which provides three sets of information: 1) early diagnostics of brain diseases, by detecting biomarkers using cell cultures, 2) the prognosis of disease progression by assessing the changes in biomarkers levels, and 3) personalized instructions on dietary, supplementary, and other approaches that could bring benefit for the individual brain cells. By combining disease detection much before the onset of visible symptoms and the individual-tailored approach, “Check My Brain” points the direction that is required in addressing the threat of neurodegenerative diseases.

## Exosomes as a new tool against dementia

As much as aging is a natural process, diseases and impairments occurring during its course are not. In Europe alone, it is expected that by 2050 the number of people living with dementia will increase by 43.85% – this imposes a large financial and health burden on modern society, warranting the development of novel experimental approaches stemming from personalized therapy – one of the most prominent being extracellular vesicles.

Extracellular vesicles (EVs) are small, membranous particles released by cells. The most widely studied EVs are exosomes. They are derived from endosomes and microvesicles, which develop from outward budding of the plasma membrane (30). The contents of exosomes are closely associated with their parent cells and include diversified proteins, lipids, noncoding RNA (including circular RNA and microRNA [miRNA]), and other molecules that may transport the exosome contents to the neighboring or more distant cells. Exosomes can contain up to 11 261 proteins, 2375 mRNA, and 764 miRNA sequences (31). Since one of the major obstacles in brain diseases is BBB impermeability, exosomes offer a unique advantage over most other approaches: they easily cross the BBB and deliver RNA sequences (32). In the central nervous system, both neurons and neuroglial cells can secrete and release exosomes into the extracellular environment, suggesting a diversified and important functions of exosomes (33). Owing to the ability of EVs to transport cargo packaged by the originating cell, their role in the pathogenesis of neurological conditions, particularly neurodegenerative diseases associated with misfolded proteins, has become a growing area of interest.

Neurodegenerative diseases share a common mechanism by which distinct proteins become misfolded and deposited in specific regions during the pathogenic process (34). After initiating factors appear within the cells, their delivery to the extracellular space is accelerated using exosomes as potential carriers. Since most tau proteins released into extracellular fluids are cut off from mid-region tau (35), it is interesting to hypothesize that a full-length tau needed for disease progression is indeed brought by exosomes. Moreover, in the plaques of patients with AD, amyloid β-peptide colocalizes with specific exosomal proteins (such as Alix), indicating that amyloid β-peptide at least in some specific cases may be deposited over exosomes secreted from neural cells in AD (36).

In addition to being used as carriers, exosomes can also be used in the diagnostics of brain diseases. The detection of exosomes in body fluids is becoming a novel tool for improving diagnosis and monitoring the biological activity of pathological process before the manifestation of apparent clinical symptoms. Indeed, toxic proteins present in the exosomes can be detected in the early stage of a variety of neurodegenerative diseases (37). On the other hand, when treatment is concerned, exosomes are potent messengers able to protect neurons from oxidative stress. Moreover, they could be used as an excellent vehicle for gene therapy, since when compared with viruses, nanoparticles, and liposomes, exosomes have better therapeutic effect, targeting ability, low immune response, and safety (38).

## Electromagnetic fields and ozone in the treatment of brain diseases

Apart from oxidative stress, imbalance in calcium homeostasis, and hormonal factors, AD is also characterized by inflammation and disorders of the cell cycle. These cause severe loss of neurons and synapses, as well as reactive gliosis, potentially altering the nature of innate electromagnetic fields (EMF) around axons (39). Recently, in order to improve age-related physiological and pathological cognitive impairments, different methods for non-invasive brain stimulation using electromagnetic fields have appeared. All of these therapies greatly rely on the main property of EMFs – the ability to influence charged cells and molecules in their vicinity.

After the success in treating anxiety and panic disorders, repetitive transcranial magnetic stimulation (rTMS) has recently become the talking point in the AD treatment. This method uses an electric pulse generator and magnetic coil placed above the scalp to apply changing magnetic fields to the desired regions in order to, through electromagnetic induction, cause or increase electrical current flow through nerve cells (40). When it comes to AD specifically, various studies have shown that rTMS is capable of modulating cortical excitability and inducing long-lasting neuroplastic changes, even after the pulse application has ended (41-43)

Similar to rTMS, transcranial direct-current stimulation applies constant low direct current via electrodes to influence membrane depolarization (44), cause changes in N-methyl-D-aspartic receptors, and initiate long-term potentiation-like mechanisms (45).

Other types of electromagnetic field therapy (EMF) are being investigated for their influence on various molecular mechanisms connected with the pathogenesis of AD, as well as innate electrical activity of the brain (46,47). One of these is deep brain stimulation, which uses implanted neurostimulators, including electrodes, and a battery source within a titanium housing. It sends electrical impulses to targeted regions in order to treat mostly movement and depressive disorders, as well as chronic pain. However, nowadays, it is also increasingly being studied with respect to the treatment of AD-related memory impairment (48,49).

Preclinical research is also being conducted on miRNA modulation using low frequency electromagnetic field (LF-EMF). Since miRNAs regulate the expression of key proteins involved in the pathogenesis of AD, these studies show interesting results in terms of the ability of LF-EMF to change miRNA expression (50). Through initiating miRNA-mediated epigenetic regulation, LF-EMF might be able to rebalance the deregulated molecular pathways occurring in patients with AD. The main molecular mechanisms behind this process involve the reduction of β-secretase – suggesting a protective function of the electromagnetic field, which would be contrary to the formation of amyloid beta (Aβ) – and an increased expression of miR-107, which is a negative regulator of BACE1 (50). Similarly, in mice models, radio frequency electromagnetic field has been shown to reduce the Aβ plaques and amyloid precursor protein (APP) in the whole brain, but its effect depended on the stage of AD (51).

However, electromagnetic field treatments are not the only innovative approaches in the field of AD diagnosis and therapy. Novel diagnostics and personalized detection techniques are being investigated, one of which is the use of continuous wave muon beams. Muons are a type of an elementary particle characterized as a lepton, carrying the same charge as an electron (−1 *e*) but with a half an integer spin. They are 207 times heavier than electrons and can, therefore, penetrate deeper into the objects or tissue while maintaining a more uniform dose (52). Bossoni et al performed a muon spin rotation experiment in patients with AD, with an aim of probing the composition of the mineral core of ferritin, a protein that stores iron (53). Since patients with AD often have altered composition of ferritin’s iron core, the authors investigated the structure of isolated ferritin proteins from a freshly frozen brain hemisphere of an AD patient and a healthy control. Interestingly, the ferritins isolated from the control patient contained a mineral compatible with ferrihydrite, while those isolated from the patient with AD contained a crystalline phase, possibly compatible with magnetite or maghemite. This additionally highlighted the importance of developing novel diagnostics and experimental tools for dementia research (53).

Another approach that has recently attracted attention is the therapeutic application of ozone. Ozone is a charged inorganic molecule that, through electrical activity, influences the phospholipids in the cell’s membrane, causing a downstream cascade of events. Different from classical single-molecule pharmacological principles, ozone generates a number of secondary compounds, which, in sub-micromolar concentrations trigger a variety of reactions (54). Apart from being known for its applications as a disinfectant, ozone has strong immunomodulatory and healing effects, which allow its use to treat wounds and pain in oral medicine (55) and to influence immune reactions with effects linked to cytokines (56). Interestingly, both our unpublished data and the recently published data by another group suggest that ozone variously influences nervous system cells (57). The most striking consequence of ozone application is its influence on APP accumulation, which improves cognitive status of mice affected by AD (57).

In conclusion, here we presented a joint call for a wide recognition of dementia as one of the largest threats for the aging human society. There is a need for a truly coordinated international action that will set up new standards: translation of innovative approaches for early diagnostic and personalized and precision therapy. Only in this way, we will enjoy the benefits of long human life and recognize healthy aging as the basic human right.
